# A young woman with dramatic pulmonary hypertension and respiratory failure: a case report

**DOI:** 10.3389/fmed.2026.1788480

**Published:** 2026-05-19

**Authors:** Min Li, Yaru Zhang, Mingjie Liu, Chunyi Lv, Dezhi Li, Yunyan Wan, Yuanyuan Sun, Ling Zhu

**Affiliations:** 1 Department of Respiratory and Critical Care Medicine, Provincial Hospital Affiliated to Shandong First Medical University, Jinan, China; 2 Department of Lung Function, Provincial Hospital Affiliated to Shandong First Medical University, Jinan, China

**Keywords:** acid *α*-glucosidase, case report, Pompe disease, pulmonary hypertension, type II respiratory failure

## Abstract

Acid *α*-glucosidase (GAA) is the sole lysosomal enzyme responsible for degrading glycogen into glucose monomers. Deficiency of GAA leads to progressive lysosomal glycogen accumulation and cellular dysfunction, resulting in Pompe disease (PD), an autosomal recessive disorder. This case report described a 41-year-old woman who presented with progressive dyspnea and decreased exercise tolerance. The preliminary examination in the outpatient clinic showed that the patient suffered from pulmonary hypertension (PH). After the patient was admitted to the hospital and further relevant auxiliary examinations were completed, and finally a diagnosis of late-onset Pompe disease (LOPD) was confirmed. Protein structure prediction suggested that the double mutation might disrupt the hydrogen bond network, hydrophobic core, and aromatic interactions of the GAA enzyme, potentially reducing the stability of the protein (ΔΔG = −2.55 kcal/mol). Although these computational insights provide a theoretical basis for the clinical phenotype, they require further validation. This is a rare case of PD complicated with PH in the absence of childhood symptoms, suggesting that specific missense mutations may contribute to late-onset and milder phenotypes. This case underscores that clinicians should strengthen clinical discrimination when diagnosing respiratory failure and PH. Understanding the characteristics of infantile-onset Pompe disease (IOPD)—relatively mild enzymatic activity impairment and late onset—helps in the clinical management of genetic metabolic.

## 1 Introduction

Acid *α*-glucosidase (GAA), a lysosomal acid hydrolase, catalyzes the hydrolysis of α-1,4 and α-1,6 glycosidic bonds in glycogen to glucose monomers, serving as the sole enzyme responsible for lysosomal glycogen degradation (1). Mutations lead to dysfunction in GAA synthesis, post-translational modification, and lysosomal trafficking/maturation, impairing enzymatic activity, promoting lysosomal glycogen accumulation, and resulting in progressive cellular dysfunction (2). Deficiency of GAA causes Pompe disease (PD), an autosomal recessive disorder, with a broad clinical spectrum ranging from severe infantile-onset to milder late-onset phenotypes depending on the degree of enzyme impairment (3).

Clinically, PD manifests as a phenotypic spectrum, classically categorized into IOPD and LOPD. Patients with IOPD exhibit absent or extremely low residual GAA activity (less than 1%), presenting within the first months of life with hypertrophic cardiomyopathy, generalized muscle weakness, and hypotonia, followed by cardiorespiratory failure and typically death within 1 year (4, 5). In contrast, LOPD patients retain 10–30% residual enzyme activity (6). Symptoms may appear at any age. Generally, they are characterized by progressive skeletal muscle dysfunction, initially involving the proximal muscles of the lower limbs and the paraspinal muscles, and then affecting the diaphragm and the accessory muscles of respiration. Eventually, respiratory failure is the main cause of death (7). However, patients with LOPD vary greatly in the age of onset and the speed of disease progression, and their clinical manifestations are also diverse. Some patients present with PH, a rare complication.

PH encompasses a heterogeneous group of disorders characterized by elevated mean pulmonary artery pressure (mPAP) exceeding the upper limit of normal at 20 mmHg by right heart catheterization (RHC) (8). PH has complex etiologies. The etiologies of PH are clinically categorized into five distinct groups based on the WHO classification, reflecting its complex and multifactorial nature (9). In recent years, the number of case reports on PD complicated by PH has been increasing. The four previously reported patients all exhibited unexplained developmental delays from infancy or childhood (10–13). However, the case we reported this time is quite special. This case underscores the importance of identifying reversible, hypoventilation-related PH in adults and suggests that specific missense mutations may contribute to late-onset phenotypes that escape early detection.

## 2 Case history

### 2.1 Case report

**Figure 1:**
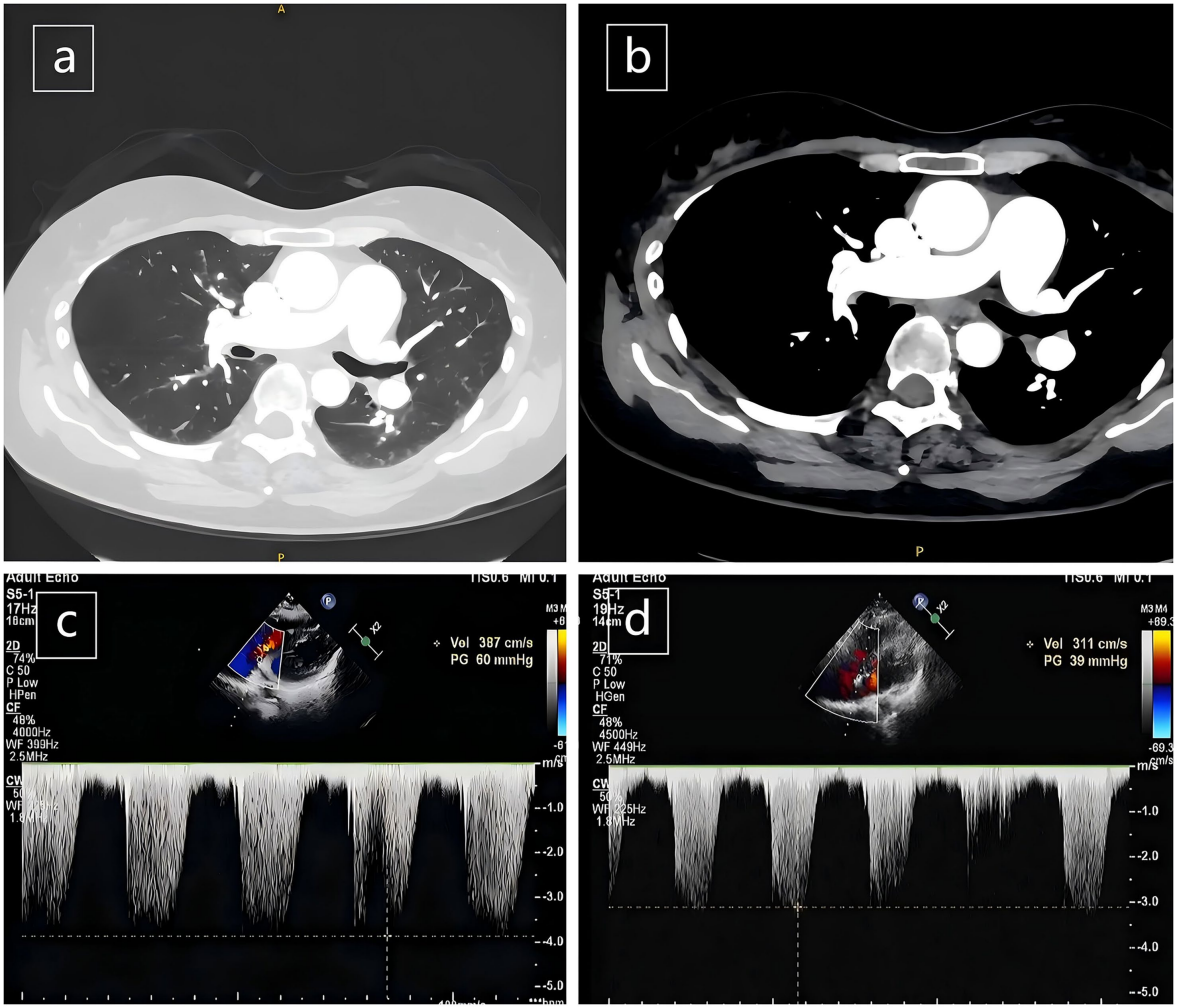
**(a)** The chest CT was largely unremarkable; **(b)** the CTPA did not detect any thrombi in the pulmonary arteries; **(c)** transthoracic echocardiography before NIPPV revealed severe pulmonary hypertension with an estimated PASP of 60 mmHg; **(d)** follow-up after NIPPV treatment demonstrated substantial regression of PASP to 39 mmHg.

The patient is a 41-year-old female. She began experiencing exertional fatigue 1 year ago, accompanied by paroxysmal coughing and exercise-induced dyspnea. Over the past month, her symptoms had been significantly aggravated, which led her to seek medical attention at our hospital. Subsequently, the chest CT showed no significant abnormal changes in the lungs (Figure 1). The transthoracic echocardiography examination revealed that the patient had PH with the pulmonary artery systolic pressure (PASP) being 65 mmHg (Figure 1) and the computed tomography pulmonary angiography (CTPA) indicated that there was no obvious abnormal embolism in the pulmonary artery (Figure 1). The pulmonary function testing suggested severe restrictive ventilatory dysfunction and the arterial blood gas (ABG) analysis showed type II respiratory failure, indicating that the patient’s dyspnea was caused by PH and respiratory failure. The comprehensive results of the patient’s physical examination and auxiliary examinations are as follows: The patient had no family history of PH, no history of exposure to drugs or toxins, and no history of traveling in epidemic areas. The patient has two children, both healthy. The virus series tests (including human immunodeficiency virus) were negative. The rheumatoid triad tests, anti-phospholipid antibody panel, vasculitis antibody panel, anti-acute ocular antibody panel, and anti-nuclear antibody panel all showed no abnormalities. Additionally, echocardiography revealed no congenital heart disease in the patient. Therefore, in addition to idiopathic pulmonary hypertension, Group 1 Pulmonary arterial hypertension (PAH) was excluded. The level of N-terminal pro-B-type natriuretic peptide (99.7 pg./mL) was not significantly elevated. The patient had no history of hypertension, coronary heart disease, heart failure, or valvular heart disease. The cardiac ultrasound revealed no signs of left heart disease, excluding group 2 PH associated with left heart disease. The CTPA did not detect any thrombi in the pulmonary arteries, and the lower limb arterial/venous Doppler examinations also found no thrombi, excluding group 4 PH associated with chronic pulmonary artery obstruction.

**Figure 2:**
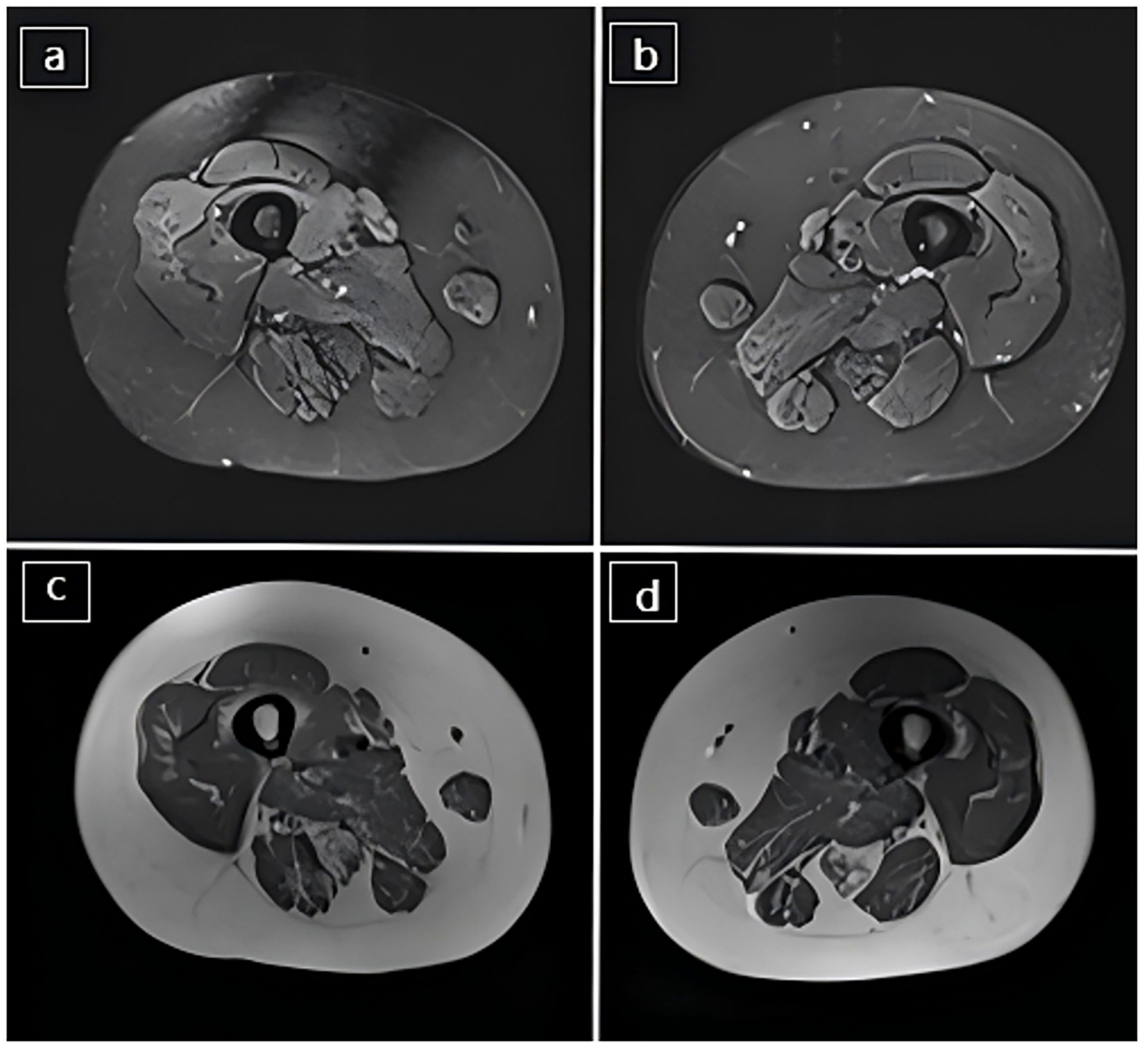
**(a)** Left thigh T2-weighted fat-suppressed image shows diffuse hyperintensity in the quadriceps and posterior thigh muscles, suggestive of active myopathic changes; **(b)** Right thigh T2-weighted fat-suppressed image with similar distribution of hyperintense signals; **(c)** Left thigh T1-weighted image shows marked fatty infiltration, especially in the quadriceps; **(d)** Right thigh T1-weighted image with extensive fatty replacement in both anterior and posterior muscle groups, reflecting chronic muscle degeneration.

Due to the patient’s difficulty in breathing, we conducted an ABG analysis as soon as she was admitted. It is worth noting that although the chest CT and CTPA appeared normal, the ABG analysis indicated the presence of type II respiratory failure (pH 7.24, PaCO₂ 115.3 mmHg, PaO₂ 215.5 mmHg, HCO₃^−^ 48.2 mmol/L, FiO₂ 37%) and extremely high partial PaCO₂ (pH 7.24, PaCO₂ 115.3 mmHg). After 2 days of non-invasive positive pressure ventilation (NIPPV) support, the ABG improved (pH 7.46, PaCO₂ 58.1 mmHg, PaO₂ 163.9 mmHg, HCO₃^−^ 40.6 mmol/L, FiO₂ 35%), point-of-care echocardiography revealed: the structure of the right heart recovered, and the PASP was normal, and after being discharged from the hospital, follow-up echocardiography performed during the outpatient revisit demonstrated: the PASP remained close to normal (Figure 1), which indicated that the patient’s PH was secondary to respiratory failure. Pulmonary function testing and chest CT results demonstrated that the type II respiratory failure was not attributable to parenchymal lung disease. The chest CT showed no significant abnormalities. Additionally, due to the patient’s lack of a long-term smoking history, chronic cough, or expectoration symptoms, excluding group 3 PH associated with lung diseases and/or hypoxia. We further investigated the cause and regarded neuromuscular diseases (including respiratory muscle weakness and neuromuscular junction-related diseases) or metabolic disorders as potential causes. Corresponding examinations were conducted. The results of electromyography (EMG) were consistent with the “muscle fiber necrosis-regeneration” dynamic changes of metabolic myopathy and the normal nerve conduction velocities (NCV) and F-wave latencies indicated that the lesion site was in the muscle tissue rather than in the peripheral nerves or neuromuscular junctions, effectively excluding myasthenia gravis (MG) and Lambert-Eaton syndrome (LES). The muscle magnetic resonance imaging (MRI) showed severe atrophy and fat infiltration in the bilateral gluteal and posteromedial thigh muscles (Figure 2). Combining the patient’s lifelong progressive proximal muscle weakness, the occurrence of respiratory failure in early adulthood, PH caused by respiratory failure, and the characteristic EMG/MRI examination results, the differential diagnosis finally focused on myopathy. PD became the primary suspect due to its characteristic triad of proximal limb weakness, respiratory muscle involvement, and restrictive lung disease. Enzymatic analysis, genetic analysis, and next-generation sequencing (NGS) confirmed the diagnosis. The activity of leukocyte GAA was significantly reduced (0.56 μmol/L/h, with a normal range of 1.29–20.34 μmol/L/h), functionally verifying lysosomal glycogen metabolism dysfunction. And the compound heterozygous pathogenic *GAA* mutations (c.2105G > A/p. Arg702His and c.2238G > C/p. Trp746Cys) genetically elucidated the phenotypic basis. Both variants were classified as pathogenic in the ClinVar database and met the molecular diagnostic criteria for PD. These missense mutations had been previously documented in relevant literature (14, 15), and the c.2238G > C/p. Trp746Cys variant was relatively common among Chinese patients with LOPD (16).

### 2.2 The intramolecular interactions among amino acids at the site of alteration

**Figure 3:**
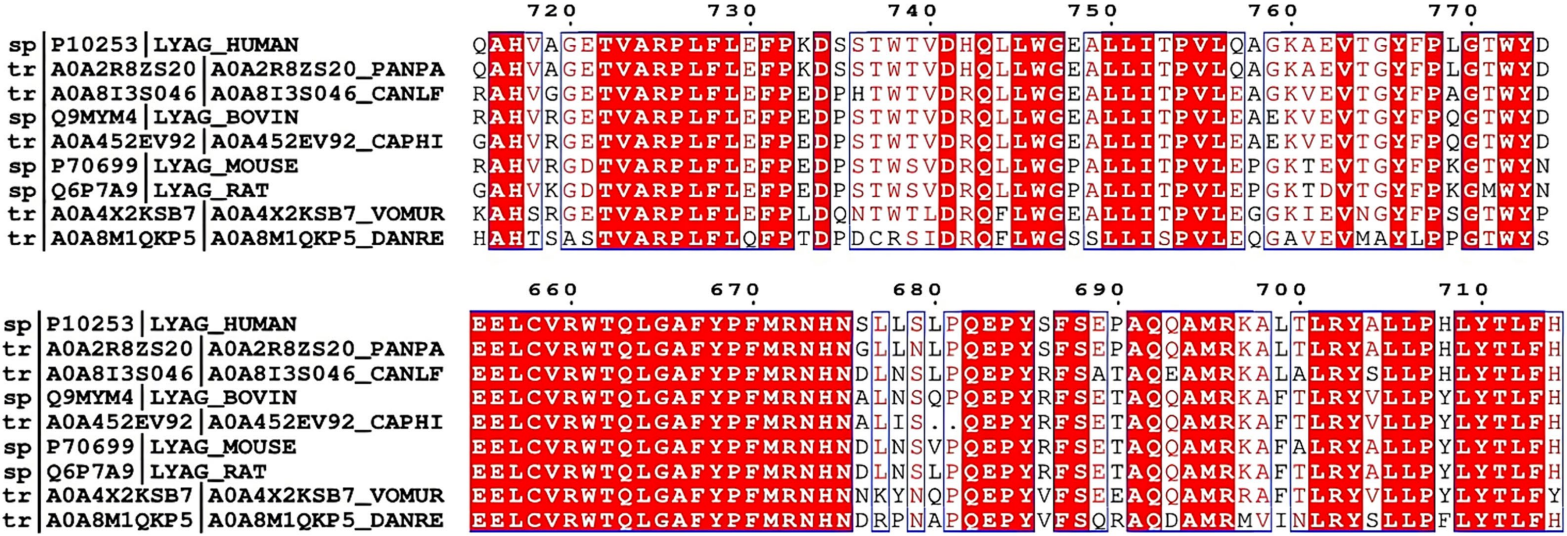
Arg702 and Trp746 are highly conserved across multiple species.

**Figure 4:**
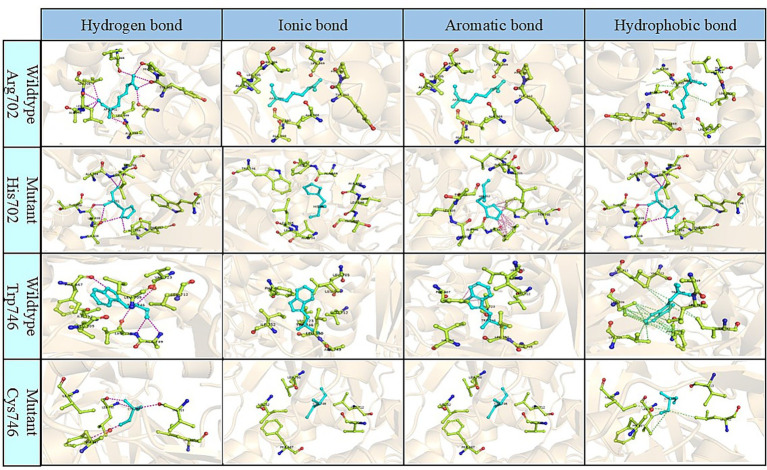
Comparison of intramolecular bonds at the alteration site in wild-type and mutant proteins, computed and visualized via DDMut. The mutations are predicted to decrease the overall stability of the protein.

We observed that Arg702 and Trp746 are highly conserved across multiple species, underscoring their critical roles in maintaining the structural and functional integrity of the GAA protein (Figure 3). To further investigate the disease mechanisms caused by Arg702 and Trp746 mutations, we utilized the machine learning tool DDMut (https://biosig.lab.uq.edu.au/ddmut/, accessed on April 15, 2025) for predictive analysis. The results revealed the following structural features in the wild-type protein: Arg702 forms a hydrogen bond network with Leu369, Ala666, Tyr668, Pro669, Ala698, Leu699, Leu705, and Leu706, as well as hydrophobic interactions involving Leu706 and Ala666. Notably, ionic or aromatic bonds were absent in these interactions. In contrast, the His702 mutant retains hydrogen bonds with Ala666, Ala698, Leu699, Leu705, and Leu706, gains aromatic interactions with Phe667 and Trp746, but loses hydrophobic interactions with Leu706. Concurrently, wild-type Trp746 stabilizes the local structure through hydrogen bonds with Phe667, Val723, Ala749, and Leu750, aromatic interactions with Phe667, and an extensive hydrophobic network encompassing Phe667, Leu705, Leu709, Leu712, Leu750, and Ile752. However, the Cys746 mutation exhibits a reduction in hydrogen bonds (retaining only Phe667, Val723, and Leu750), complete loss of aromatic interactions, and diminished hydrophobic interactions limited to Leu712, Leu750, and Ile752. The double MT (His702 + Cys746) is predicted to lead to a drastic reduction in hydrogen bonds, disruption of the critical hydrophobic core, and irreversible loss of aromatic interactions at Trp746, potentially decreasing protein stability (Figure 4).

### 2.3 The prediction of protein stability

The value of ΔΔG^stability^ of these mutations was calculated using DDMut, utilizing the experimentally determined structure of wild-type human GAA (PDB ID#8CB1) as control. The ΔΔG^stability^ for the two mutations, Arg702His and Trp746Cys, were −2.68 kcal/mol and −1.88 kcal/mol, respectively. The combined ΔΔG^stability^ for the double mutation was calculated as −2.55 kcal/mol, indicating that these mutations synergistically destabilize the protein structure.

### 2.4 Follow-up

The patient’s initial transthoracic echocardiography performed on February 25, 2025, revealed severe pulmonary hypertension with an estimated PASP of 60 mmHg. After treatment with NIPPV, the final in-hospital follow-up on March 17, 2025, showed that cardiac ultrasound demonstrated normal right ventricular structure and function. Furthermore, the patient reported a marked reduction in dyspnea and a significant improvement in daily activity tolerance. After discharge, regular telephone follow-ups were conducted, during which the patient reported a stable clinical condition, with no recurrence of symptoms or discomfort such as chest tightness, dyspnea, or shortness of breath. Clinical guidelines emphasize that GAA should be initiated as soon as skeletal or respiratory muscle involvement is confirmed to slow disease progression (17). While NIPPV can rapidly reverse secondary PH, ERT remains vital for stabilizing long-term respiratory function. Research suggests that ERT can improve or stabilize forced vital capacity (FVC) and muscle strength, potentially preventing the deterioration of ventilatory drive that leads to PH recurrence (18). Despite these clinical benefits and our repeated persuasion, the patient ultimately declined this treatment modality due to personal factors.

## 3 Discussion

This case illustrates an unusual clinical presentation of LOPD, where type II respiratory failure and secondary PH served as the initial manifestations, in the absence of any childhood neuromuscular symptoms. Such atypical phenotypes highlight the limitations of conventional diagnostic paradigms and emphasize the need to broaden the clinical lens when evaluating adult patients with unexplained respiratory dysfunction.

In our patient, compound heterozygous pathogenic mutations in the GAA gene (c.2105G > A/p. Arg702His and c.2238G > C/p. Trp746Cys) were identified, both of which have been classified as pathogenic by the PD GAA Variant Database (http://www.pompevariantdatabase.nl). The pathogenic mechanism of c.2105G > A has been confirmed by multiple studies: on one hand, this variant co-occurs with the clinical phenotype of typical LOPD in several cases (14, 19); on the other hand, *in vitro* expression experiments have shown that the protein conformational changes caused by this variant significantly affect precursor processing and enzyme maturation. In COS-7 cell experiments, the intracellular enzyme activity retains only about 13%, while the extracellular secretory activity is even lower, at merely 5% (20). Moreover, the c.2238G > C mutation is one of the most common GAA gene variants in LOPD patients in mainland China (21). Although both of these variants have been reported individually in previous studies, their occurrence as a set of compound heterozygous mutations is a rare clinical case. This specific combination likely allows residual enzyme activity to maintain metabolic homeostasis. Consequently, glycogen metabolism disorders remain at a subclinical level before puberty. This explains why the patient did not exhibit typical movement disorders during childhood but instead presented with PH and respiratory failure in adulthood.

Currently, only a few cases have suggested that PD, PH, and respiratory failure can coexist, and the underlying mechanisms among the three remain unclear. A notable feature of this case is that the patient presented with both PH and respiratory failure upon consultation, and the PASP returned to normal rapidly after the respiratory failure was corrected. This suggests that the PH secondary to chronic alveolar hypoventilation and hypercapnia rather than primary pulmonary vascular disease. This mechanism is consistent with previous reports (13) on LOPD-PH patients. In these cases, involvement of the diaphragm and accessory respiratory muscles leads to chronic hypoventilation. This hypercapnic state then induces pulmonary artery constriction, ultimately resulting in PH. Studies (22, 23) have shown that hypercapnia can enhance the pulmonary vascular constrictive response to hypoxia while alleviating structural remodeling. This indicates that the development of PH accompanied by hypercapnia is largely due to functional regulatory disorders rather than simple vascular stenosis. Clinically, existing studies have confirmed that early initiation of NIPPV treatment can effectively correct hypercapnia, significantly improve PASP, oxygenation function, and exercise tolerance, further supporting the reversibility of PH in hypoventilation-related diseases (24). The rapid improvement of PH with NIPPV support in this case also emphasizes again that potential ventilatory disorders should be identified early in patients with PH complicated by hypercapnia, and timely intervention should be given.

Despite its clinical significance, this study has several limitations. First, RHC was not performed in this specific patient. At the time of presentation, the patient was in a life-threatening acute type II respiratory failure state. A clinical diagnosis of Group 3 PH was established, and after the initiation of NIPPV support, echocardiography demonstrated rapid and significant normalization of the PASP. Therefore, further RHC was not pursued. Second, the follow-up duration in this study is relatively short; although the current follow-up data confirm the immediate effectiveness of NIPPV, longer-term monitoring is required to assess the durability of this reversal. Regarding long-term management, ERT has not been initiated due to the patient’s personal factors. Finally, although the protein structure analysis provides a theoretical basis for the pathogenicity of the observed variants, these in silico predictions still require further experimental validation. Therefore, implementing a comprehensive management strategy remains essential to optimize the patient’s long-term prognosis.

## 4 Conclusion

Through a multi-dimensional correlation analysis integrating clinical phenotype, genotype, and protein function, this case systematically elucidates the mechanisms underlying LOPD complicated by PH and respiratory failure. The clinical practice in this case demonstrates that early NIPPV support can effectively reverse LOPD-PH and improve prognosis. Future studies are required to further clarify the pathological mechanisms of *GAA* mutations and explore potential targeted therapies for patients with GAA deficiency.

## Data Availability

The original contributions presented in the study are included in the article/supplementary material, further inquiries can be directed to the corresponding author/s.
